# Orofacial granulomatosis associated with Crohn’s disease: clinical phenotype and response to biologics and Janus kinase inhibitor therapy in a large multi-center cohort

**DOI:** 10.1093/ecco-jcc/jjag139

**Published:** 2026-09-21

**Authors:** Robert D Lees, Priyanka Patel, Gregory Young, Lauren Beck, Angelica Molinillo Marin, Daniel Parkinson, Laetitia Pele, Miles Parkes, Jeremy Sanderson, Cameron Herbert, Esther Hullah, Christopher A Lamb, Joel Mawdsley, Richard Ally Speight

**Affiliations:** Translational and Clinical Research Institute, Newcastle University, Newcastle upon Tyne, United Kingdom; Department of Gastroenterology, The Newcastle upon Tyne Hospitals NHS Foundation Trust, Newcastle upon Tyne, United Kingdom; Department of Gastroenterology, Guy’s and St Thomas’ NHS Foundation Trust, London, United Kingdom; Translational and Clinical Research Institute, Newcastle University, Newcastle upon Tyne, United Kingdom; Translational and Clinical Research Institute, Newcastle University, Newcastle upon Tyne, United Kingdom; Department of Gastroenterology, Guy’s and St Thomas’ NHS Foundation Trust, London, United Kingdom; Department of Cellular Pathology, The Newcastle upon Tyne Hospitals NHS Foundation Trust, Newcastle upon Tyne, United Kingdom; NIHR BioResource for Translational Research, Cambridge, United Kingdom; Department of Haematology, University of Cambridge, Cambridge, United Kingdom; NIHR BioResource for Translational Research, Cambridge, United Kingdom; Department of Gastroenterology, Cambridge University Hospitals NHS Foundation Trust, Cambridge, United Kingdom; Department of Gastroenterology, Guy’s and St Thomas’ NHS Foundation Trust, London, United Kingdom; Department of Oral Medicine, Guy's and St Thomas’ NHS Foundation Trust, London, United Kingdom; Department of Oral Medicine, Guy's and St Thomas’ NHS Foundation Trust, London, United Kingdom; Translational and Clinical Research Institute, Newcastle University, Newcastle upon Tyne, United Kingdom; Department of Gastroenterology, The Newcastle upon Tyne Hospitals NHS Foundation Trust, Newcastle upon Tyne, United Kingdom; Department of Gastroenterology, Guy’s and St Thomas’ NHS Foundation Trust, London, United Kingdom; Translational and Clinical Research Institute, Newcastle University, Newcastle upon Tyne, United Kingdom; Department of Gastroenterology, The Newcastle upon Tyne Hospitals NHS Foundation Trust, Newcastle upon Tyne, United Kingdom

**Keywords:** orofacial granulomatosis, biologics, JAK inhibitors

## Abstract

**Background & Aims:**

Orofacial granulomatosis (OFG) is strongly associated with Crohn’s disease (CD). The CD phenotype associated with OFG is poorly defined, and there is little evidence regarding treatment. We aimed to characterize OFG-associated CD phenotypes and evaluate the response of OFG to advanced therapies in the largest cohort described to date.

**Methods:**

We conducted a retrospective cohort study of patients with OFG and CD from two UK centers, derived from patient records compiled since 2008. Phenotypic characteristics were compared with a CD control cohort of 1609 individuals without OFG from the NIHR IBD-BioResource. Clinical response to biologic/Janus kinase inhibitor (JAKi) therapies was assessed.

**Results:**

A total of 117 patients were included. Compared with controls, OFG was associated with earlier CD diagnosis (<17 years: 43% vs 22%, *P* < .001), increased ileocolonic disease (61% vs 41%, *P* < .001), perianal disease (46% vs 35%, *P* = .012), and upper gastrointestinal disease (21% vs 8%, *P* < .001). Clinical remission was achieved in 29 of 53 (55%) prescribed infliximab and 35 of 59 (59%) prescribed adalimumab, with frequent dose escalation. Remission was achieved with ustekinumab, risankizumab, and upadacitinib in 14 of 25 (56%), 6 of 12 (50%), and 3 of 5 (60%), respectively in biologic-experienced patients. Higher anti-TNF drug levels were associated with higher odds of achieving treatment response (adalimumab OR 1.14 per 1 µg/mL increase, *P* = .049; infliximab OR 1.27, *P* = .047).

**Conclusions:**

OFG is associated with a distinct CD phenotype characterized by frequent UGI and perianal involvement. Advanced therapies, including anti-TNF, anti-IL23, and JAKi therapies, are effective, with evidence supporting dose optimization.

## Graphical abstract

**Graphical Abstract:**
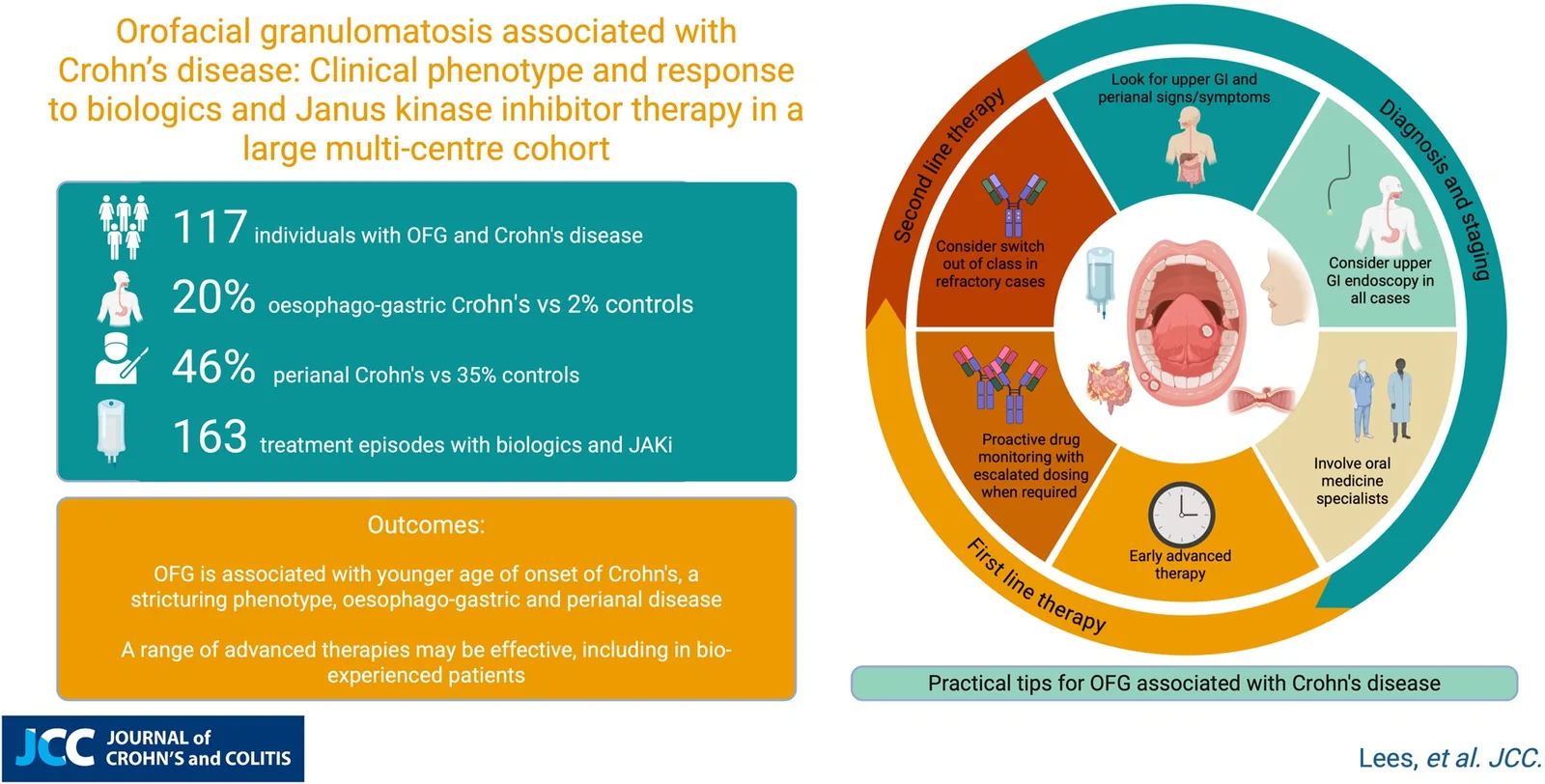

## 1. Introduction

**Figure 1.**
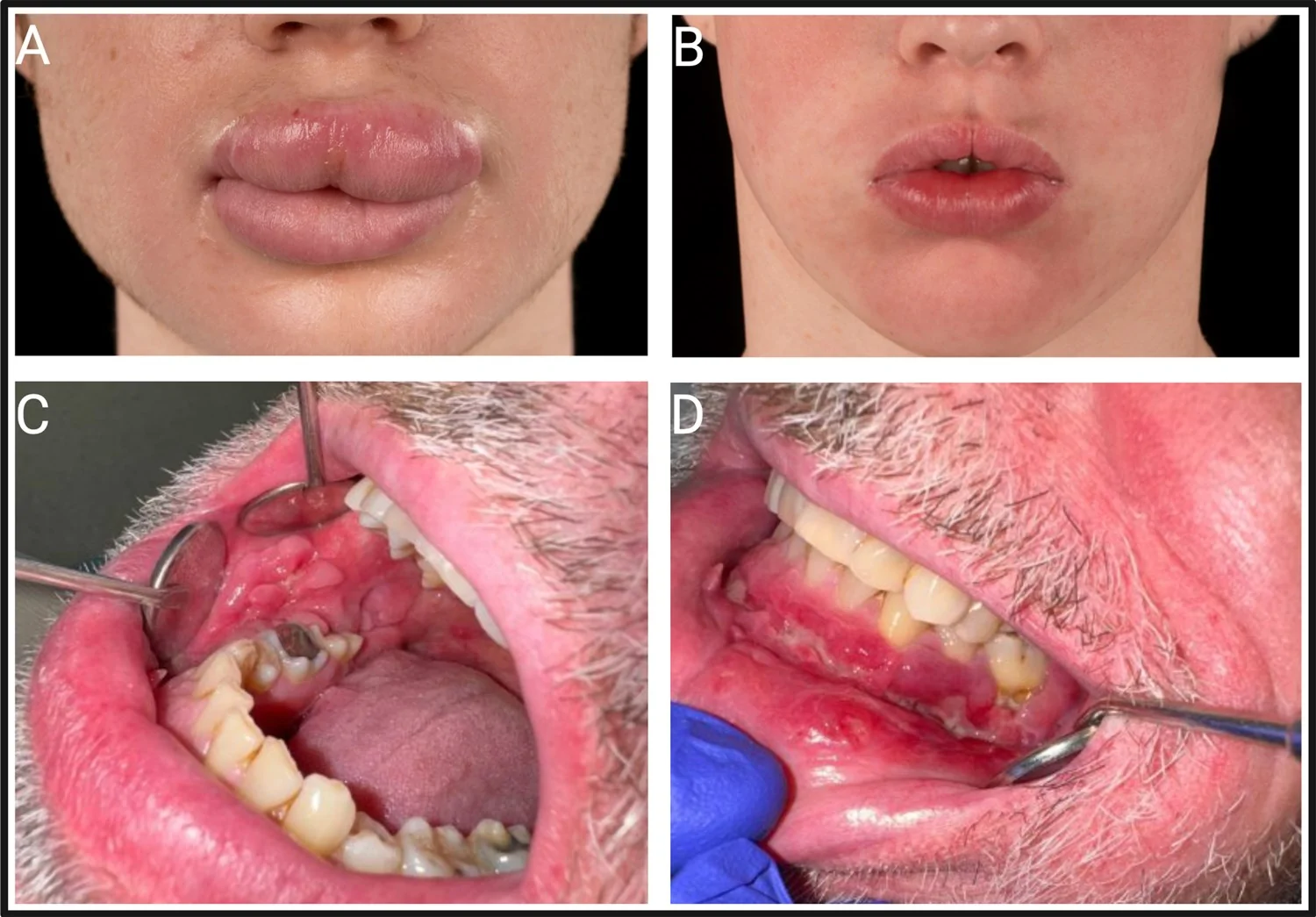
Features of OFG including labial swelling (A) and fissuring (B), cobblestoning of the buccal mucosa (C), and sulcal ulceration (D).

Orofacial granulomatosis (OFG) is an uncommon, relapsing remitting, chronic inflammatory condition of the face and mouth, characterized by swelling of the lips, tongue fissuring, cobblestoning of the buccal mucosa, and intra-oral ulceration (Figure 1). The condition is predominantly diagnosed in children and young adults, however the etiology is poorly defined, with several dietary antigens and other environment exposures having been implicated.^1–3^

Although OFG may present in isolation, there is a strong association between OFG and Crohn’s disease (CD).^4^^,^^5^ Approximately 50% of individuals with OFG also have, or will develop, CD.^4–7^ Where OFG and CD co-exist, there is no clear consensus regarding whether the orofacial symptoms represent a separate disease process or an oral manifestation of CD.^5^^,^^7^ It has been suggested that a “posterior pattern” of disease with predominant buccal and sulcal ulceration is more commonly associated with CD, while an “anterior pattern” with predominant lip involvement is associated with isolated OFG.^8^

Attempts to further elucidate this relationship through characterization of the CD phenotype associated with OFG have been hampered by small numbers in uncontrolled cohorts.^9^^,^^10^ Phillips et al.^9^ have published an uncontrolled case series of 28 individuals with concurrent CD and OFG, reporting upper gastrointestinal tract (UGI) involvement in 6 of 28 patients and perianal disease in 11 of 28, suggesting that these phenotypes may be over-represented, albeit with small numbers. Establishing the phenotype of CD associated with OFG in a larger cohort may have important implications for disease staging, for instance, through greater utilization of UGI endoscopy, and the therapeutic strategies employed.

Morbidity associated with OFG is high and management is often complex.^11^ While dietary and topical therapies are commonly utilized, in cases of concurrent CD, oral symptoms may fail to respond and advanced therapies (biologics and small molecules) are employed.^11^ However, the evidence base for efficacy of advanced therapies in the treatment of OFG is extremely limited. The largest case series of individuals receiving anti-tumor necrosis factor (anti-TNF) therapy for OFG, published by Elliott et al.^12^, contains just 14 cases, reporting short-term clinical response in 10 of 14 (71%) individuals managed with infliximab. Subsequently, Phillips et al.^9^ published a multi-center case series of 28 patients, supported by the European Crohn’s and Colitis Organization, and performed as part of the Collaborative Network of Exceptionally Rare case reports (CONFER) project.^9^ The authors reported clinical remission of OFG in 9 of 14 (64%) individuals treated with anti-TNF agents.^9^

With early advanced therapy increasingly becoming standard of care in CD due to its association with superior long-term outcomes, there is a clear need to establish an evidence base to guide the use of biologics and small molecules in the OFG cohort.^13^ Currently, beyond anti-TNF, the evidence base for ustekinumab, vedolizumab, and Janus kinase (JAK) inhibitors is limited to individual case reports, and to the authors’ knowledge, there are no reported cases of use of anti-IL23 agents for OFG.^14–17^

To address these gaps in our current knowledge, we have assembled the largest published cohort of OFG associated with CD from 2 large referral centers. Using routinely collected data, we aimed to characterize the CD phenotype associated with OFG in comparison to a site-specific control cohort. We further aimed to evaluate OFG response in the largest study of advanced therapies for OFG to date, including, to the authors’ knowledge, the first reported cases of anti-IL23 therapy for OFG.

## 2. Methods

### Cohort selection

A cohort of individuals with established diagnoses of both OFG and CD was assembled by clinicians at 2 large tertiary referral centers: The Newcastle upon Tyne Hospitals NHS Foundation Trust (NUTH) and Guy’s and St Thomas’ NHS Foundation Trust (GSTT). Patients were identified through existing departmental registers, routine clinical encounters and cross-referencing of patients recruited to the NIHR IBD BioResource. Only patients with established diagnoses of both OFG and CD confirmed by IBD and/or oral medicine specialists were eligible for inclusion. Cases of OFG diagnosed clinically or histologically were eligible for inclusion, in line with widespread diagnostic practice and previous studies.^18^^,^^19^ To facilitate inclusion of patients prescribed recently licensed advanced therapies, no minimum follow-up period was specified. All identified patients were included if their clinical care included time within the era of the electronic patient record (2008 onward).

Aggregate phenotyping data was extracted from the NIHR IBD BioResource for 1609 individuals with CD, who did not have a recorded diagnosis of OFG, recruited to the IBD BioResource from the 2 study sites, forming a site-specific control cohort. The IBD BioResource is a national research platform of over 50 000 participants with IBD.^20^ During the recruitment period, all patients with CD at the study sites were eligible for recruitment, forming a large cohort which is representative of the CD phenotypes and standard of care encountered at these centers. Detailed phenotyping, including Montreal classification and presence of OFG, but not OFG features or treatment response, is recorded at the time of recruitment to the IBD BioResource.

### Data extraction and synthesis

All data were extracted from patients’ electronic medical records between November 2025 and March 2026 and pseudoanonymized by members of their clinical teams. Data were extracted according to a pre-specified proforma and collated on Microsoft Excel. Data extracted included age and sex, age at OFG and CD onset, OFG features described, oral histology results, Montreal classification of disease, previous UGI endoscopy results, surgical history, presence/absence of extra-intestinal manifestations (EIMs), and the date of last review by the gastroenterology or oral medicine team. Features of OFG were further categorized into anterior signs (lip swelling, facial swelling, angular cheilitis) and posterior signs (intra-oral ulceration, cobblestoning of the buccal mucosa, gingivitis). Upper GI CD was defined, in line with the Montreal classification, as endoscopic and/or histological evidence of inflammation proximal to the terminal ileum, diagnosed as CD by the responsible IBD specialist.^21^ We also noted cases of upper GI CD which involved the oesophagus and/or stomach specifically. Perianal disease was defined as any history of perianal ulcer, fissure, or fistula diagnosed as perianal CD by the patient’s IBD specialist.

History of treatment with biologic agents and small molecules licensed for treatment of CD was also recorded, including sequencing of these therapies. Where possible, clinical response of OFG to advanced therapies, as assessed by an IBD or oral medicine specialist, was recorded for each treatment episode where there was documentation of clinical features of active OFG prior to initiation of the treatment. Treatment episodes which pre-dated the first presentation of OFG or were initiated only for management of luminal disease (ie, when no OFG symptoms were present prior to treatment initiation) were not included in the treatment response analysis. Whether patients were prescribed the standard licensed dose or an escalated maintenance dosing regimen was noted. Escalated dosing was defined as dosing above infliximab 5 mg/kg 8 weekly, adalimumab 40 mg 2 weekly, ustekinumab 90 mg 8 weekly, risankizumab 360 mg 8 weekly, vedolizumab 300 mg 8 weekly, or upadacitinib 30 mg daily. As there is no validated scoring system for OFG, response was classified as 1 of 4 pre-specified outcomes: clinical remission (defined as complete clinical remission of OFG symptoms), partial response (defined as improvement in OFG symptoms, but not remission), no response or unknown (where no clinical response was documented). All treated patients were included in the analysis of response rates to each drug, including those with unknown response. If patients received the same drug across multiple treatment episodes (>12 months apart), response was assessed for the first treatment episode.

Drug levels obtained during routine clinical care (first treatment episodes with infliximab and adalimumab only) were captured. We recorded the highest trough drug level for infliximab. Adalimumab levels taken at any point during the maintenance phase of treatment were accepted as drug levels reach steady state in this scenario.^22^ Levels above the upper limit of detection (>20 µg/mL) were recorded as 20 µg/mL. Drug level monitoring for other advanced therapies is not routinely performed in our centers and these data were consequently not available.

### Statistical analysis

Statistical analysis was performed in R (version 4.5.2, visualization packages: ggplot2, ggalluvial, ggsankey). Univariate statistical comparison was made between cohorts for each component of the Montreal classification using Fisher’s exact test. If data were not available for one component, the patient was excluded from analysis of that component only. Disease behavior was analyzed using logistic regression models with adjustment for duration of disease (time in years from diagnosis to last clinical review), which was included as a continuous covariate to account for differences in time at risk of developing stricturing or penetrating disease. Adjusted odds ratios with 95% CIs were estimated for each outcome.

Drug levels were analyzed both as continuous variables and as pre-defined categorical strata (<5, 5–10, 10–15, and >15 µg/mL). Clinical response was treated as an ordered outcome (no response, partial response, or clinical remission). Patients without exposure to the relevant drug or without available drug level data were excluded. Associations between drug level and response were assessed using ordinal logistic regression, with effect estimates expressed as ORs for improvement in response category. Nonparametric tests (Kruskal–Wallis and Spearman rank correlation) were used to assess differences and trends across response groups. For categorical analyses, Fisher’s exact test was used to evaluate associations due to small cell counts, and ordinal logistic regression was used to estimate relative odds across level categories, with the lowest category (<5 µg/mL) as the reference.

### Ethical statement

Patient records were accessed only by members of the patients’ clinical team. Each patient was assigned a pseudo-identifier and no patient identifiable data was collected. Patient records were accessed at Guy’s and St Thomas’ NHS Foundation Trust in line with existing ethical approval (REC 12/YH/0172). Caldicott approval was sought and granted from the Newcastle upon Tyne Hospitals NHS Foundation Trust.

## 3. Results

### Cohort characteristics and phenotype

A total of 117 patients with established diagnoses of OFG and CD were included in the analysis (71 NUTH, 46 GSTT). No cases of OFG associated with ulcerative colitis or IBD-unclassified were identified. The median length of follow up was 12 years (range 0-49 years). Sixty-six of 117 patients (56%) were male. Seven hundred and seventy-two of 1609 controls (48%) were male, and the median length of follow-up was 11 years (range 0-60 years).

The median age of OFG diagnosis was 24 (IQR 19.0), and the median age of CD diagnosis was 18 (IQR 11.0). The diagnostic sequence was established in 108 of 117 cases: OFG was diagnosed prior to CD in 27 of 108 (25%), OFG and CD were diagnosed concurrently in 35 of 108 (33%), and OFG was diagnosed after CD in 46 of 108 (43%).

**Figure 2.**
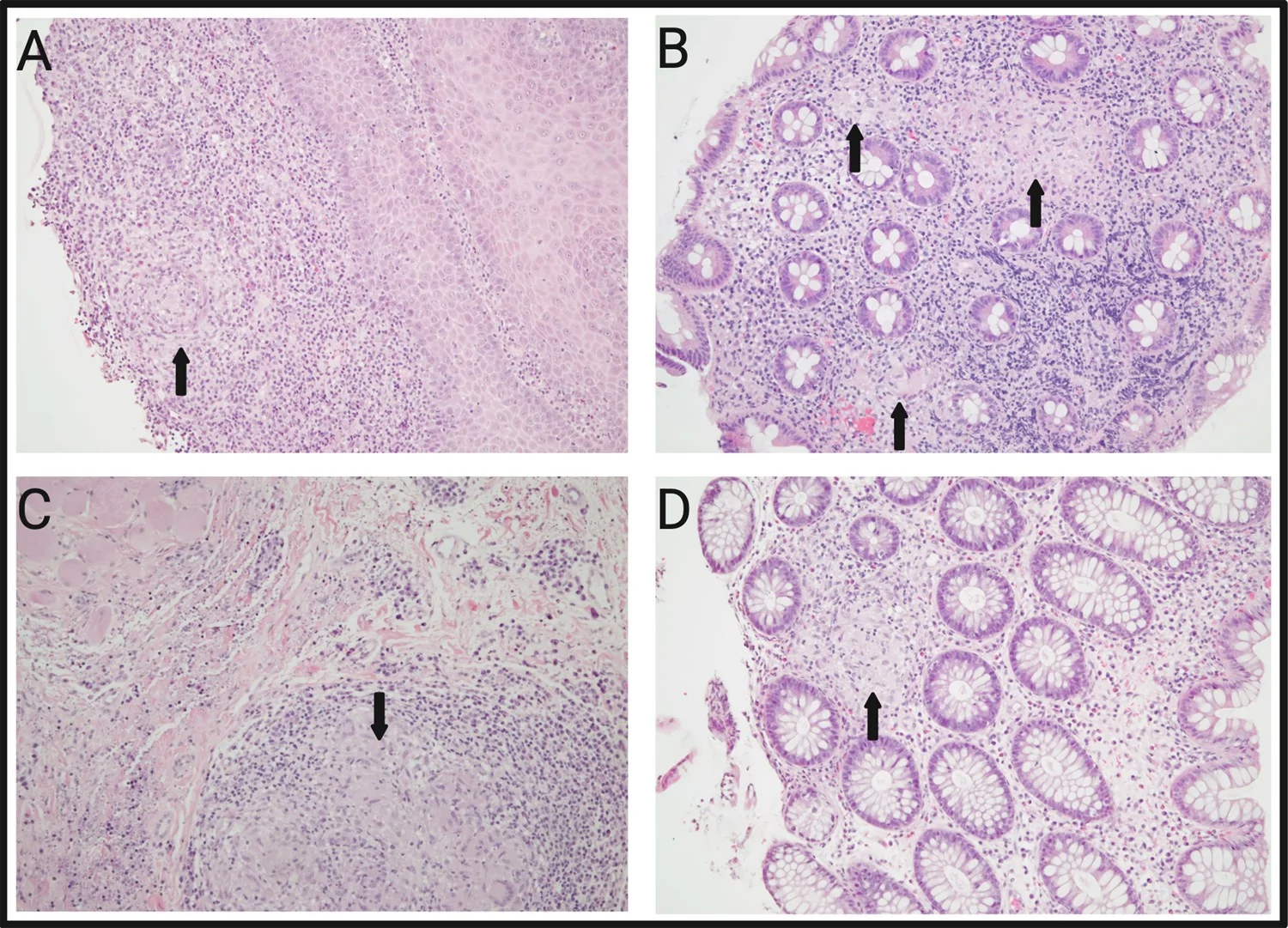
Hematoxylin and eosin staining of paired retromolar oral mucosal (A) and colonic biopsies (B), and lip (C) and colonic biopsies (D) in 2 patients with OFG and Crohn’s disease. Black arrows indicate granulomas.

The most common reported OFG clinical features were lip swelling (72%), intra-oral ulceration (50%), cobblestoning of the buccal mucosa (40%), angular cheilitis (29%), and gingivitis (12%). Anterior signs only were reported in 11%, posterior signs only in 13% and a combination of anterior and posterior signs in 76%. Histological examination of oral biopsies was undertaken in 33 cases, which revealed granuloma in 26 of 33 cases (79%) (Figure 2).

Montreal classification of the OFG and control cohorts is outlined in Table 1. Compared to controls, patients with OFG were more likely to be diagnosed with CD <17 years of age (43% vs 22%, *P* < .001), and less likely to be diagnosed >40 years of age (5% vs 16%, *P* = .001).

**Table 1.** Crohn’s disease phenotype of OFG and control cohorts.

	OFG cohort *n* (%)	Control cohort *n* (%)
**Age at diagnosis**		
**≤16 (A1)**	49 (43)	360 (22)
**17-40 (A2)**	59 (52)	987 (62)
**>40 (A3)**	6 (5)	258 (16)
**Disease location**		
**Terminal ileal (L1)**	17 (15)	443 (29)
**Colonic (L2)**	22 (19)	400 (26)
**Ileocolonic (L3)**	68 (60)	659 (43)
**Isolated upper GI (L4)**	1 (1)	24 (2)
**Esophago-gastric involvement**	24 (21)	32 (2)
**Isolated perianal disease**	5 (4)	12 (1)
**Disease behavior**		
**Non-stricturing, non-penetrating (B1)**	56 (57)	949 (64)
**Stricturing (B2)**	36 (37)	345 (23)
**Penetrating (B3)**	6 (6)	198 (13)

Percentages represent proportion of those for whom data were available within each domain (OFG cohort age at diagnosis *n* = 114, disease location *n* = 113, disease behavior *n* = 98).

OFG patients had a lower prevalence of terminal ileal (L1) disease (15% vs 29%, *P* = .001), but a higher prevalence of ileocolonic (L3) disease (61% vs 41%, *P* < .001). OFG was also associated with a higher prevalence of perianal CD (46% vs 35%, *P* = .012), including a higher prevalence of isolated perianal disease (4% vs 1%, *P* = .005).

Twenty-five of 54 (46%) OFG patients who underwent upper GI endoscopy at any point received a diagnosis of upper GI CD. 23 of 54 (43%) had esophago-gastric involvement and 9 of 54 (17%) had an oesophageal stricture. Of 27 patients to undergo upper GI endoscopy in the absence of any upper GI symptoms, 8 of 27 (30%) had evidence of sub-clinical upper GI CD. Compared to controls, patients with OFG were more likely have a diagnosis of upper GI (L4) disease (21% vs 8%, *P* < .001), which was driven by high rates of oesophago-gastric disease (20% vs 2%, *P* < .001). There was no association between OFG features (anterior, posterior, or combination) and presence of UGI CD (*P* = .51).

OFG was independently associated with stricturing (B2) disease. After adjustment for disease duration, cases had approximately 2-fold higher odds of B2 disease compared with controls (OR 1.99, 95% CI 1.29-3.03; *P* = .0015). In contrast, there was no evidence of an association between OFG and penetrating (B3) disease (OR 0.66, 95% CI 0.30-1.26; *P* = .24).

Forty-four percent of the OFG cohort had undergone resectional surgery for CD. Including perianal surgery, there was no significant difference in the rate of surgery for CD (50% OFG vs 56% controls). Twenty-four of 117 OFG patients (21%) had at least 1 EIM. The most commonly reported EIMs were inflammatory arthritis including enteropathic arthritis and axial spondyloarthropathy (8 cases), genital cutaneous CD (6 cases), and psoriasis (5 cases). Control data were available for rates of rheumatological EIMs with no significant difference observed.

### Response to advanced therapies

**Figure 3.**
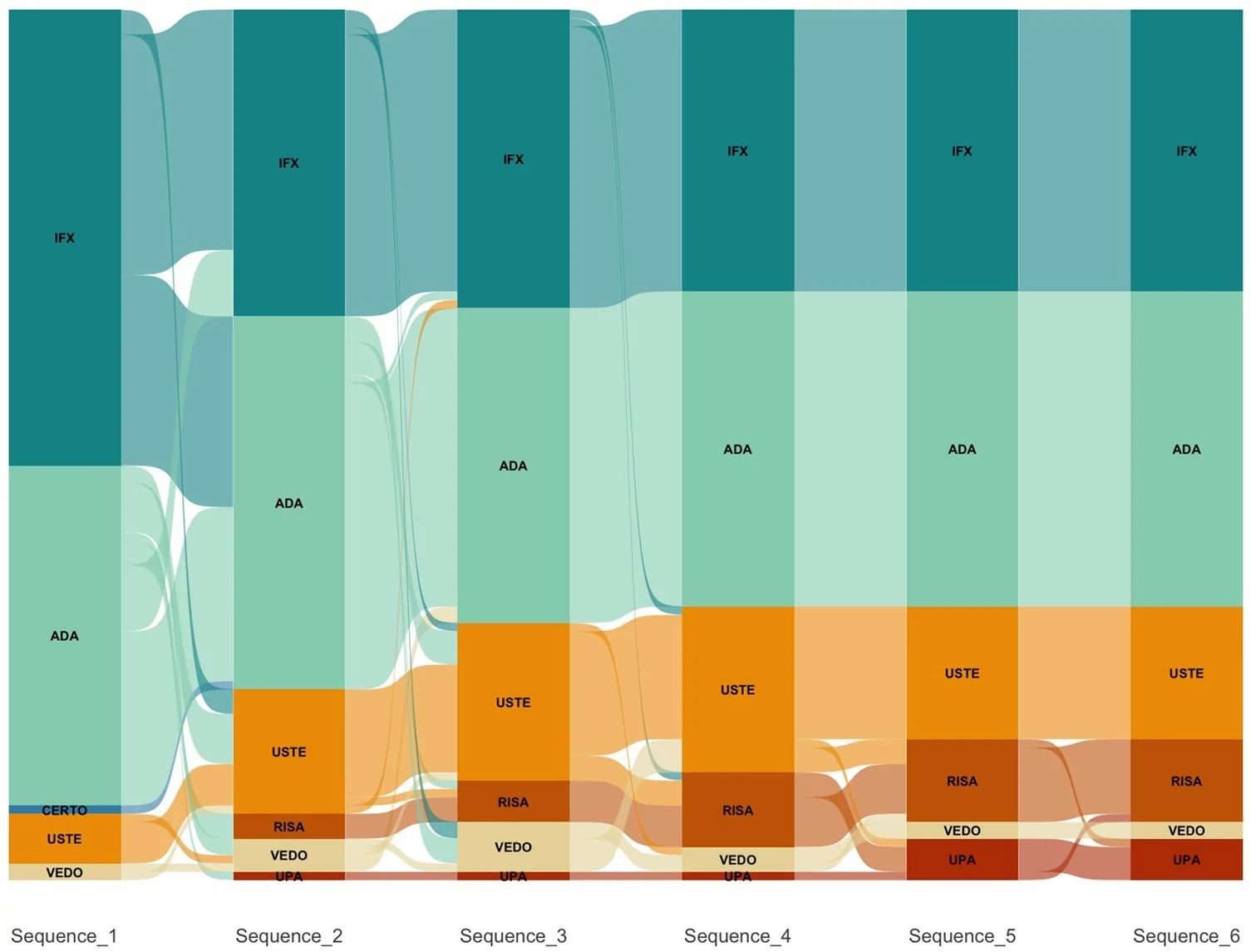
Sequencing of biologic and small molecule therapies in 105 patients with OFG and Crohn’s disease, showing up to first 6 treatment episodes. IFX = infliximab; ADA = adalimumab; CERTO = certolizumab; USTE = ustekinumab; RISA = risankizumab; VEDO = vedolizumab; UPA = upadacitinib.

One hundred and five of 117 patients (90%) received at least 1 advanced therapy while they had signs or symptoms of active OFG. The mean number of advanced therapies was 1.4 (*SD = *1.23). One hundred and sixty-three treatment episodes were assessed (53 infliximab, 59 adalimumab, 1 certolizumab, 25 ustekinumab, 12 risankizumab, 8 vedolizumab, and 5 upadacitinib). Median time from treatment initiation to response assessment was 6 months (IQR 3 months). Sequencing of advanced therapies is outlined in Figure 3.

Ninety-seven of 105 (92%) received an anti-TNF agent as first-line advanced therapy. A similar proportion of patients achieved clinical remission with infliximab (55%) and adalimumab (59%). Of those to achieve clinical remission, 38% of those receiving infliximab and 29% of those receiving adalimumab did so on an escalated dosing regimen.

Most patients who received ustekinumab or risankizumab did so following exposure to 1 or more anti-TNF agent (Figure 3). Despite utilization in a biologic-experienced group, patients achieved clinical remission with ustekinumab in 56% of cases and risankizumab in 50% of cases.

**Figure 4.**
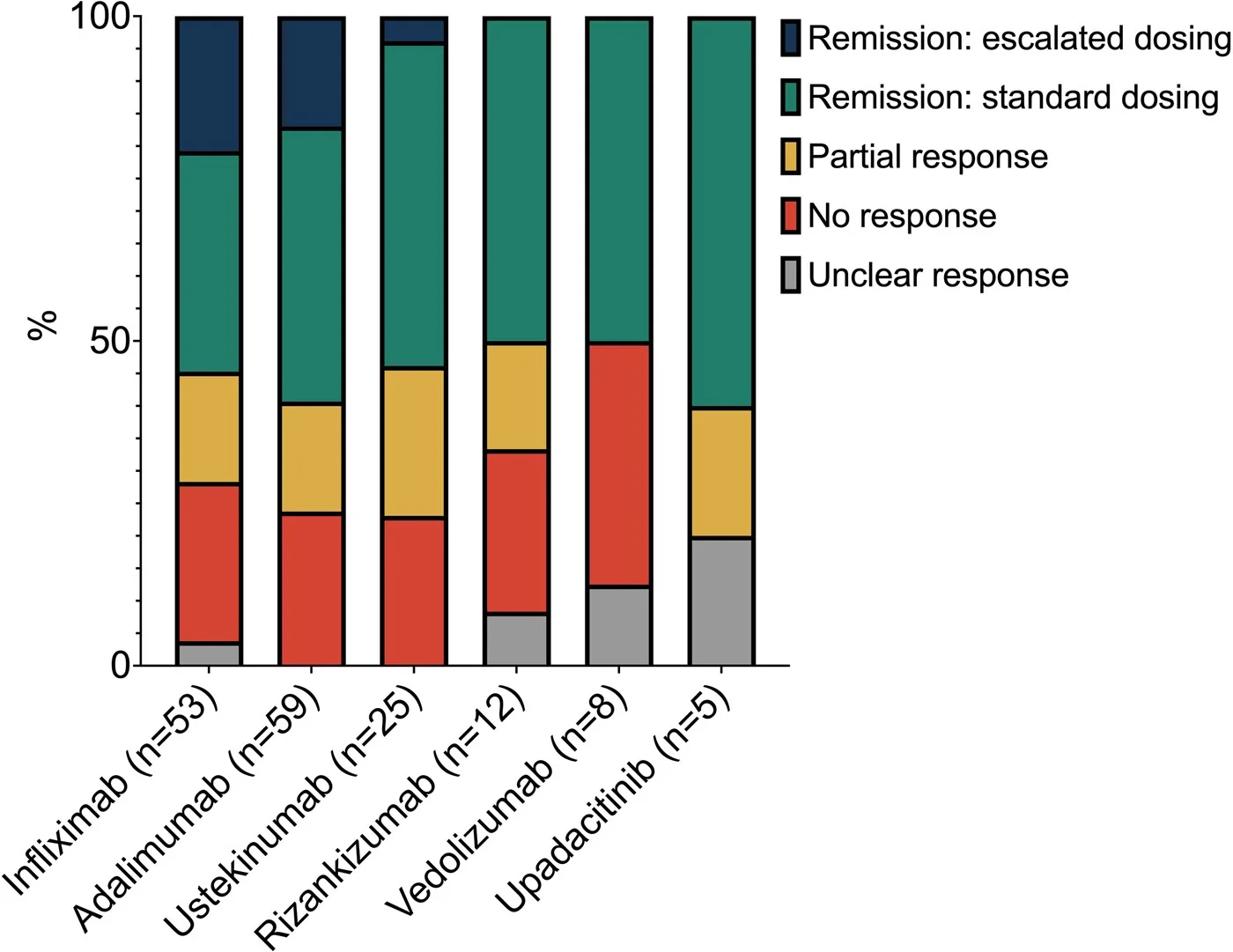
Response of OFG to biologic and Janus kinase inhibitor therapies.

A small cohort of patients were prescribed vedolizumab, although 4 of 8 (50%) achieved clinical remission of OFG. Five patients were prescribed upadacitinib with 3 (60%) achieving clinical remission in a biologic-experienced group. Clinical response of OFG to advanced therapies is outlined in Figure 4.

One hundred and six of 117 patients (91%) received a thiopurine at any point, either as monotherapy or in combination with a biologic agent. Response of OFG to thiopurines could not be independently assessed, although 99 of 106 patients (93%) who received a thiopurine did so either in combination with a biologic agent, or required an advanced therapy subsequent to a period of thiopurine monotherapy, either for active OFG, active luminal disease, or both.

### Therapeutic drug monitoring and OFG response

**Figure 5.**
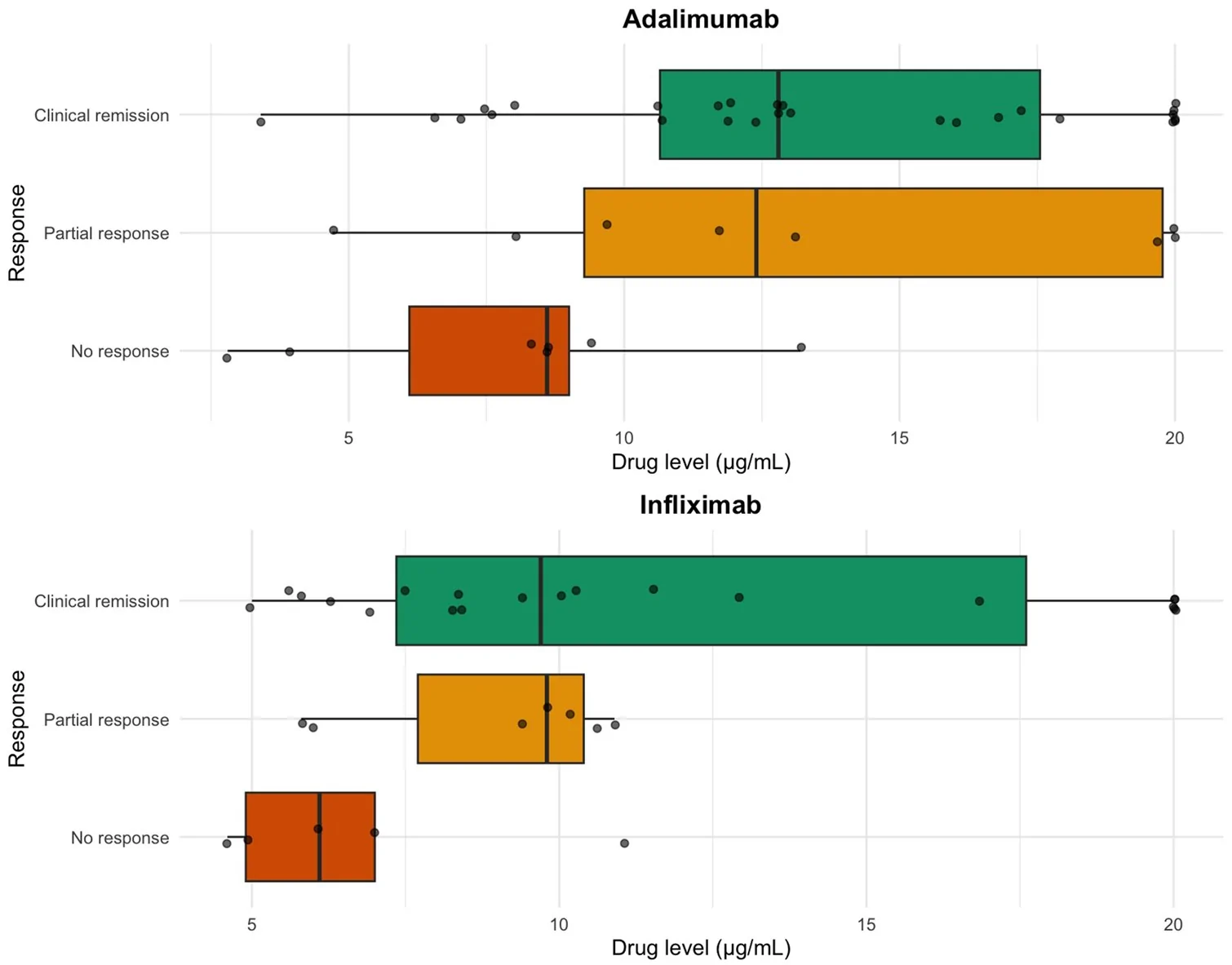
Box plots showing highest recorded drug levels for adalimumab (*n* = 42) and infliximab (*n* = 32) categorized by level of clinical response of OFG.

Among patients treated with adalimumab who had drug levels available for analysis (*n* = 42), higher drug levels were associated with improved clinical response (Figure 5). In continuous analysis, increasing adalimumab level was associated with higher odds of achieving a better response category (OR 1.14 per unit increase, 95% CI 1.01-1.31, *P* = .049). This was supported by a positive, although borderline, trend in nonparametric analyses (Spearman ρ = 0.28, *P* = .068; Kruskal–Wallis *P* = .068). When categorized, a stepwise increase in remission rates was observed across adalimumab level strata (25% for <5 µg/mL, 45% for 5-10 µg/mL, 77% for 10-15 µg/mL, and 79% for >15 µg/mL), with significantly higher odds of improved response at levels 10-15 µg/mL (OR 11.0, 95% CI 1.16-135.0, *P* = .042) and >15 µg/mL (OR 13.2, 95% CI 1.41-160.5, *P* = .028) compared with <5 µg/mL. An overall ordinal trend across categories was observed (Spearman ρ = 0.41, *P* = .0067), although Fisher’s exact test did not reach significance (*P* = .067).

Among patients treated with infliximab (*n* = 32), higher trough drug levels were also associated with improved response (OR 1.27 per unit increase, 95% CI 1.04-1.69, *P* = .047) (Figure 5). A positive but borderline trend was observed in nonparametric testing (Spearman ρ = 0.32, *P* = .071), while the Kruskal–Wallis test was not significant (*P* = .14). In categorical analyses, remission rates increased from 0% at <5 µg/mL to 100% at >15 µg/mL, but this pattern was less consistent across intermediate categories. Fisher’s exact test showed borderline evidence of association (*P* = .059), and an ordinal trend across categories was observed (Spearman ρ = 0.36, *P* = .043). However, ordinal logistic regression estimates for infliximab categories were unstable due to sparse data.

## 4. Discussion

In this large, multi-center cohort study, we provide the most comprehensive characterization to date of CD associated with OFG, alongside the largest evaluation of treatment response to biologic and JAK inhibitor therapies in this population. Our findings offer important insights into disease phenotype and provide clinically relevant real-world evidence to guide management strategies.

This represents, to our knowledge, the largest published cohort of patients with concurrent OFG and CD, substantially exceeding the size of previously reported case series, which have typically been limited to fewer than 30 individuals.^9^^,^^12^ The scale of this cohort allows for more accurate description of phenotype and treatment response, addressing a key limitation of the existing literature. Importantly, our study also includes a large, site-specific control cohort derived from the IBD BioResource, enabling direct comparison of CD phenotype between individuals with and without OFG.^20^ This represents a significant methodological advance over prior uncontrolled cohorts, where interpretation has been limited by the absence of an appropriate comparator group.^9^^,^^12^

A key finding of this study is the over-representation of UGI and perianal CD in patients with OFG. Nearly half of patients who underwent upper GI endoscopy had evidence of UGI involvement, with a predominance of oesophago-gastric disease, and a substantial proportion developing oesophageal strictures. Even when considering the entire cohort, including those who did not undergo endoscopic assessment, the prevalence of oesophago-gastric CD was markedly higher than in controls. Similarly, perianal disease was significantly more common in the OFG cohort compared to the control population. These findings are consistent with, but considerably extend, previous smaller case series suggesting enrichment of these phenotypes in OFG-associated CD.^9^

The observation that inflammation frequently involved the UGI tract adds weight to the hypothesis that OFG represents part of the Crohn’s disease spectrum, rather than a distinct extraintestinal entity.^4–7^ We did not observe any division between those with an “anterior pattern” and “posterior pattern” of clinical signs: most patients had a combination of anterior and posterior signs and there was no association between pattern of OFG symptoms and CD phenotype. Furthermore, the preponderance of stricturing disease and high rates of UGI and perianal involvement suggest that OFG may be associated with a distinct and aggressive disease subtype.

From a clinical perspective, our findings have important implications for disease assessment and monitoring. Notably, a substantial proportion of patients with no upper GI symptoms were found to have endoscopic evidence of UGI CD. This raises the possibility that subclinical UGI involvement may be under-recognized in this population. Given the observed burden of oesophageal disease, including stricturing complications, these data support consideration of routine upper GI evaluation in patients presenting with OFG, even in the absence of symptoms. Early identification of UGI disease should facilitate timely initiation of advanced therapy, with the potential to prevent complications such as fibrostenotic oesophageal disease.

In addition to phenotypic characterization, this study provides the largest dataset to date evaluating response of OFG to advanced therapies. The majority of patients were treated with anti-TNF agents as first-line therapy, reflecting current clinical practice. We demonstrate that anti-TNF therapy is effective in inducing remission of OFG in a substantial proportion of patients, consistent with prior smaller series.^9^^,^^12^ However, a notable proportion of patients achieved remission on escalated dosing regimens, which may have been initiated for oral and/or luminal disease, suggesting that OFG may be relatively refractory to standard dosing regimens. This observation is clinically relevant, as it supports a proactive approach to dose optimization in patients with persistent orofacial disease activity.

These observations correlate with our analysis of anti-TNF drug levels. We report a consistent exposure–response relationship for both adalimumab and infliximab, with higher drug levels associated with improved clinical outcomes. The agreement in direction across multiple analytical approaches, despite some tests not reaching significance, suggests that limited power rather than absence of effect likely explains borderline results. Nevertheless, this remains the first analysis of anti-TNF levels in OFG, in the largest published dataset. The stronger signal observed in categorical analyses, particularly for adalimumab, may reflect clinically meaningful threshold effects, supporting a proactive approach to dose optimization to achieve levels >10 µg/mL. It is acknowledged, however, that not all tests reached statistical significance and these findings would be strengthened by validation in larger cohorts.

Importantly, we extend the evidence base beyond anti-TNF therapy. This study includes, to our knowledge, the first systematic evaluation of IL-12/23 and IL-23 inhibition in OFG. Both ustekinumab and risankizumab were associated with encouraging rates of clinical remission, despite being used almost exclusively in biologic-experienced patients. These findings are particularly notable given that existing evidence for these agents in OFG has been limited to isolated case reports.^9^^,^^15^ Our data suggest that targeting the IL-23 pathway is an effective therapeutic strategy in CD-associated OFG, consistent with the broader efficacy of these agents in luminal CD. The responsiveness of OFG to these therapies supports the hypothesis that orofacial manifestations share common immunological mechanisms with intestinal disease. This further reinforces the concept of OFG as part of the CD disease spectrum, rather than a separate inflammatory condition.

Although numbers were smaller, encouraging response rates to JAK inhibition were also observed, despite typically being employed in a refractory and biologic-experienced cohort. Remission of OFG was also observed in 4 of 8 patients who received vedolizumab, although any benefit in this cohort is likely mediated through an improvement in luminal disease, as mucosal addressin cell-adhesion molecule-1 (MAdCAM-1), which is integral to vedolizumab mechanism of action, is not thought to be expressed in the oral mucosa.^23^ However, the limited sample size precludes definitive conclusions regarding the relative efficacy of these agents, and further study is required. Additionally, we have not appraised the efficacy of nonsystemic treatments and close collaboration with oral medicine specialists remains crucial for effective management of the condition.

This study has several limitations. First, its retrospective design introduces the potential for selection bias and incomplete data capture, particularly with respect to treatment response, which was dependent on documentation in clinical records. Furthermore, in the absence of a validated scoring system for OFG, assessment of response is limited to broad categories (no response, partial response and remission) and dependent on the physician’s global assessment, which contains a degree of subjectivity and may be vulnerable to bias. Second, upper GI endoscopy was not performed systematically in all patients, which may have led to underestimation or overestimation of the true prevalence of UGI disease. Comparison with the control group may be confounded as the proportion of controls who underwent upper GI endoscopy is unknown. Nevertheless, the finding of a high rate of UGI involvement even among asymptomatic individuals suggests that the true burden may be substantial, despite the potential for surveillance bias. Third, although the use of a site-specific control cohort with similar length of follow up is a strength, differences in follow-up duration, treatment exposures and disease severity between cohorts may confound comparisons, although this is mitigated by adjustment for disease duration in our analysis of disease behavior. Fourth, the effect of historical systemic steroid exposure, thiopurine monotherapy, topical and dietary therapies could not be independently assessed in this cohort. Finally, treatment allocation was not randomized, and confounding by indication cannot be excluded when interpreting treatment response.

**Figure 6.**
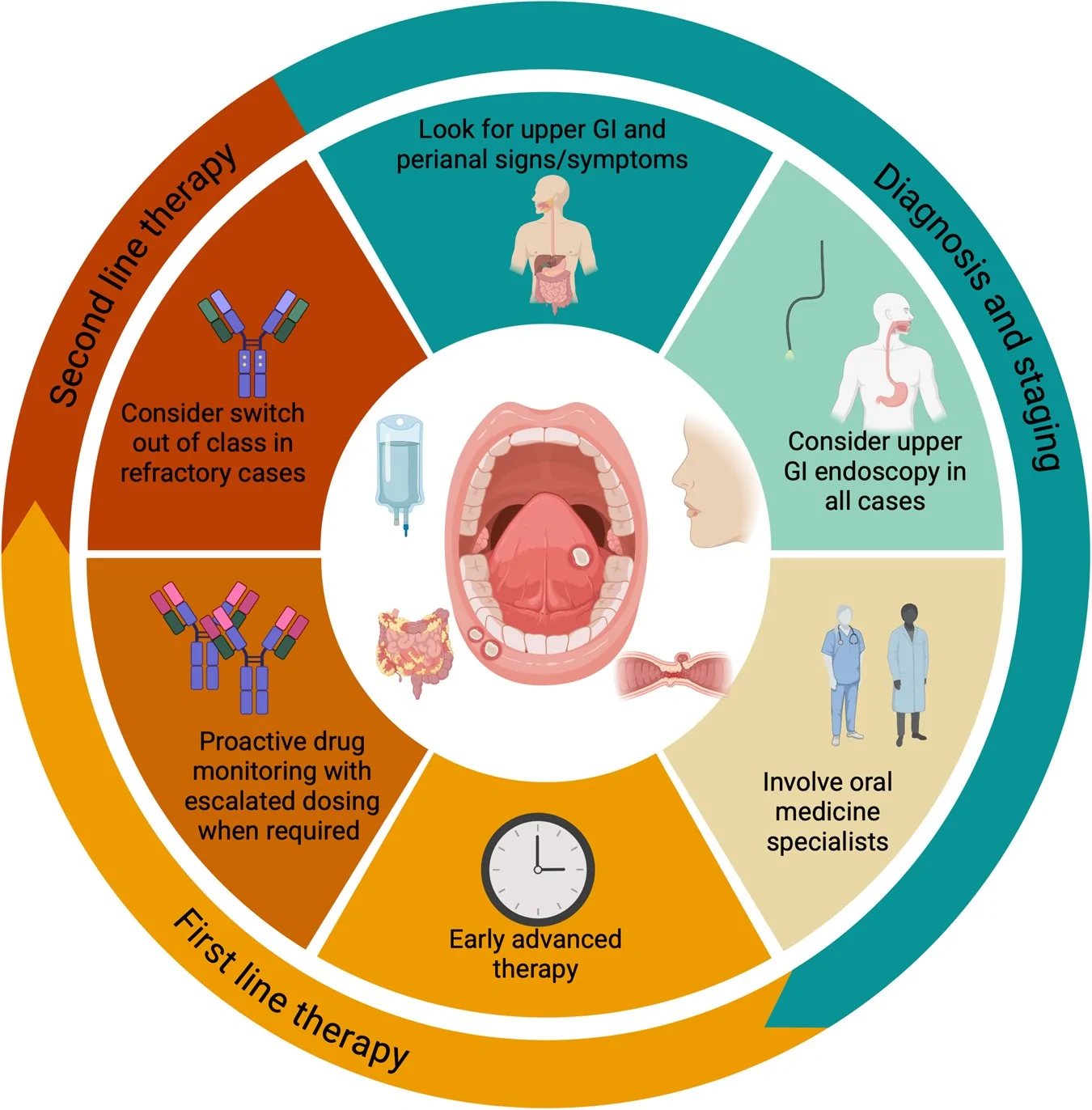
Practical tips for managing concurrent OFG and Crohn’s disease.

Despite these limitations, this study provides important advances in our understanding of OFG-associated CD which can be readily translated into practical advice for clinicians (Figure 6). The identification of a distinct phenotype characterized by high rates of UGI and perianal involvement has direct clinical relevance, supporting a more comprehensive approach to disease assessment. The demonstration of efficacy of across multiple drug classes provides a much-needed evidence base to inform therapeutic decision-making in this challenging condition.

In conclusion, OFG is strongly associated with a distinct and potentially more aggressive CD phenotype, characterized by frequent upper GI and perianal involvement and high rates of stricturing disease. These findings support the concept of OFG as part of the Crohn’s disease spectrum. Routine evaluation for upper GI disease should be considered in patients with OFG, even in the absence of symptoms, to enable early intervention. Anti-TNF therapy remains an effective first-line treatment, although dose optimization may be required, while other biologic therapies licensed for management of CD, in addition to JAK inhibitors, are also efficacious options. Further prospective studies are warranted to refine screening strategies and optimize treatment algorithms in this population.

## Data Availability

All aggregate-level data underpinning this article are included in the article. Patient-level data cannot be shared to protect the privacy of the individuals involved.
